# Activity of Combinations of Antioxidants and Anthelmintic Drugs against the Adult Stage of *Schistosoma mansoni*

**DOI:** 10.1155/2020/8843808

**Published:** 2020-08-06

**Authors:** Maria João Gouveia, Paul J. Brindley, Fátima Gärtner, Nuno Vale

**Affiliations:** ^1^Department of Molecular Pathology and Immunology, Institute of Biomedical Sciences Abel Salazar (ICBAS), University of Porto, Rua de Jorge Viterbo Ferreira 228, 4050-313 Porto, Portugal; ^2^Center for the Study in Animal Science, University of Porto (CECA/ICETA), Rua de D. Manuel II, Apartado 55142, 4051-401 Porto, Portugal; ^3^Department of Microbiology, Immunology & Tropical Medicine, Research Center for Neglected Diseases of Poverty, School of Medicine & Health Sciences, George Washington University, Washington, DC 20037, USA; ^4^Institute of Molecular Pathology and Immunology of the University of Porto (IPATIMUP), Rua Júlio Amaral de Carvalho 45, 4200-135 Porto, Portugal; ^5^i3S, Instituto de Investigação e Inovação em Saúde da Universidade do Porto, Rua Alfredo Allen 208, 4200-135 Porto, Portugal; ^6^OncoPharma Research Group, Center for Health Technology and Services Research (CINTESIS), Rua Dr. Plácido da Costa, 4200-450 Porto, Portugal; ^7^Faculty of Medicine, University of Porto, Al. Prof. Hernâni Monteiro, 4200-319 Porto, Portugal

## Abstract

Schistosomiasis remains a major neglected tropical disease. The treatment and control of schistosomiasis rely on a single drug, praziquantel (PZQ). Despite its efficacy, treatment with PZQ presents some major drawbacks including an inability of the chemotherapy to reverse disease-induced fibrosis and the prospect of the emergence of drug resistance. Here, we investigated a novel therapeutic approach with antioxidant biomolecules in combination with PZQ against the adult developmental stage of *Schistosoma mansoni* and oviposition *in vitro*, given that this therapeutic approach achieved synergistic/additive activity against larval schistosomes. The antioxidants curcumin and oxadiazole *per se* exhibited antischistosomal activity against adult worms leading to severe morphological alterations and death. Additionally, the antioxidant flavone combined with vandetanib or imatinib improved antischistosomal activity against adult forms. By contrast, however, these antioxidant-anthelmintic combinations were not as effective against adults in comparison to larval schistosomes. Nevertheless, the antioxidants alone or combined with drugs inhibited oviposition.

## 1. Introduction

Schistosomiasis is one of the major neglected tropical diseases. The infectious agents are helminth parasites of genus *Schistosoma* [1] of which three main species, *Schistosoma mansoni*, *S. haematobium*, and *S. japonicum*, are responsible for human schistosomiasis. Infection with *S. haematobium* is responsible for ~90% of cases in sub-Saharan Africa and is considered carcinogenic by the International Agency for Research on Cancer (IARC) [1]. Despite the control strategies to block transmission, schistosomiasis present elevated incidence in sub-Saharan Africa, East Asia, and Brazil affecting >200 million people and ~800 million others at risk of infection [1]. Recently, the transmission of schistosomiasis Haematobia has reemerged in southern Europe [2, 3].

For more than 40 years, the control and treatment of schistosomiasis rely on a single drug, praziquantel (PZQ), that mainly targets the parasite and not the disease sequelae [4]. Despite its efficacy against all forms of human schistosomiasis and low toxicity, the drug has major drawbacks including a limited effect on juvenile schistosomes and eggs, liver and spleen lesions as a consequence of infection and liberation of eggs by the adult worms [5]. Moreover, due to the extensive and long-term repeated use of PZQ, there is a legitimate concern about the development of drug resistance or reduced susceptibility [4]. Thus, there is a consensus on the urgent need to develop novel, affordable, and effective therapies against this debilitating neglected tropical disease. Strategies such as drug repurposing and/or combination of biologically active agents with distinct modes of action might reduce the time and cost of drug research and development [6, 7]. In our view, novel therapeutic approaches should not only focus on the elimination of the parasite but also ameliorate the pathologies associated with the infection. During the host immune response against parasite, the liberation of reactive oxygen species (ROS) might disturb the cellular antioxidant homeostasis of affected organs [8, 9]. Regarding *S. haematobium* infection, reactive electrophilic compounds, including estrogen-like metabolites, might initiate infection-associated malignancy [10, 11]. Hypothetically, using biomolecules with antioxidant properties might contribute to ameliorate the liberation of ROS, restore organ function, and/or prevent the formation of carcinogenic metabolites. Some antioxidants present promising biological properties and are considered pharmacologically safe [12, 13]. They might prevent DNA damage [14], block carcinogenesis [15], and display antischistosomal activity [16–18]. These properties flag them as potential antischistosomal drugs.

**Figure 1:**
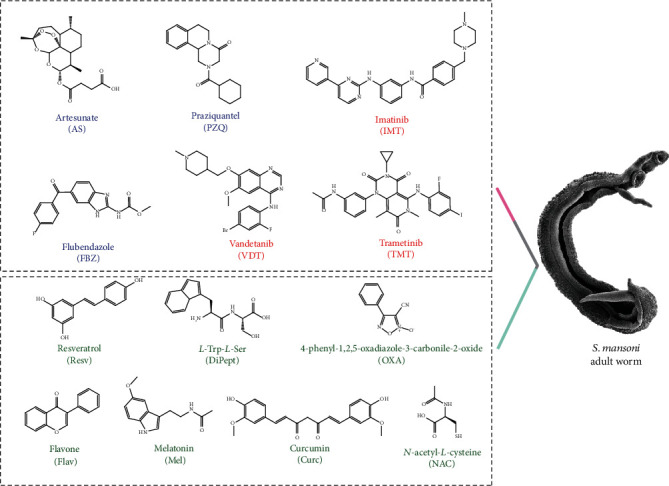
Structures of anthelminthic drugs, anticancer drugs, and antioxidant biomolecules evaluated either alone or combined against *S. mansoni* adult worms. The anthelmintic drugs are depicted in blue, anticancer drugs in red, and antioxidant biomolecules in green. PZQ: praziquantel; FBZ: flubendazole; AS: artesunate; DiPept: *L*-Tyr-*L*-Ser; Resv: resveratrol; Mel: melatonin; Flav: flavone; NAC: *N*-acetyl-*L*-cysteine; Curc: curcumin; OXA: 4-phenyl-1,2,5-oxadiazole-3-carbonile-2-oxide.

Therefore, we propose a novel therapeutic approach based on drug repurposing and a combination of a different class of drugs (anthelmintic and anticancer) with antioxidants (Figure 1) that ideally should present a dual mode of action: (1) eliminate the parasite and (2) ameliorate the infection-associated pathologies. We speculate that this novel therapeutic approach might contribute to the treatment of schistosomiasis. Previously, this novel therapeutic approach was evaluated on the newly transformed schistosomula of *S. mansoni* [17, 18]. Here, we evaluated these drugs and antioxidant combinations against the adult stage of *S. mansoni* and on oviposition by the schistosomes *in vitro*.

## 2. Materials and Methods

### 2.1. Chemicals and Culture Media

Praziquantel (PZQ), 4-phenyl-1,2,5-oxadiazole-3-carbonile-2-oxide (OXA), *N*-acetylcysteine (NAC), flavone (Flav), and flubendazole (FBZ) were purchased from Merck Sigma-Aldrich (Lisboa, Sigma), resveratrol (Resv) from Santa Cruz Biotechnology (Heidelberg, Germany), artesunate (AS), vandetanib (VDT), curcumin (Curc), and melatonin (Mel) from Cayman Chemical (Ann Arbor, MI, USA), and the dipeptide H-*L*-tryptophan-*L*-serine-OH (H-Trp-Ser-OH, DiPept) from Bachem (Bubendorf, Switzerland). The culture medium RPMI 1640 and supplements including penicillin (10.000 U/mL)/streptomycin (10 mg/mL) were from Merck Sigma-Aldrich and heat-inactivated fetal bovine serum (FBS) from Lonza (Basel, Switzerland). For *in vitro* assays, stock solutions of 2-5 mg/mL were freshly prepared in 100% dimethylsulfoxide (DMSO) (Sigma-Aldrich) and stored at 4°C. These stock solutions were diluted in fresh culture medium before its addition to the well-containing adult worms.

### 2.2. Parasites

The life cycle of the *S. mansoni* strain is maintained by passage through *Biomphalaria glabrata* snails and CD1 mice obtained from the Center for Vector and Infectious Disease Studies Francisco Cambournac and maintained at the animal facility of Public Health Care Dr. Gonçalves Ferreira (INSA-Porto). Female mice CD1 (8 weeks old) were infected with 160-180 cercariae using a tail immersion technique [19], and after 7-8 weeks of infection, *S. mansoni* adult worms were recovered under aseptic conditions by perfusion of the livers and mesenteric veins [20]. The worms were washed in RPMI 1640 medium (Merck Sigma-Aldrich), supplemented with 1% penicillin/streptomycin and 10% fetal bovine serum (FBS). The experiments were conducted following the law DL 113/2013 of the Portuguese Republic and European Directive 2010/63/UE and were approved by the Ethics of Animal Experiments of INSA-Porto (project no. 04/2018) and Directorate General Food and Veterinary.

### 2.3. *In Vitro* Antischistosomal Activity

The procedure to evaluate the antischistosomal activity of compounds alone or combined against adult worms was previously described [7]. Briefly, one pair of adult *S. mansoni* worms *in copula* (one female and one male) in RPMI 1640 medium (1 mL) was placed in each well of the 48-well plate (Nunclon, Denmark). It was evaluated one pair of worms per assay and was performed 3 assays, *n* = 3, per treatment. The screening of test compounds was performed at a concentration of 100 *μ*M and a combination at a constant ratio (1 : 1) at the same concentration. All the compounds were prepared as described above and added to RPMI 1640 medium containing the worms after a period of 24 h to recover from the eventual stress of perfusion and adaptation to culture medium. The parasites were maintained for 72 h in a constant temperature incubator at 37°C in an atmosphere of 5% CO_2_ in the air and monitored 1, 17, 24, 48, and 72 h for motor activity, mortality, and morphological alterations as described [7, 21]. Adult worms incubated with the highest concentration of DMSO (0.1%) served as a negative control. Eggs released by pair of adult worms were counted previously after the addition of drugs and antioxidants either alone or combined and after the addition of compounds (1, 24, 48, and 72 h). Phenotypic changes were recorded using a light microscope (Nikon Phase Contrast 2, LDW 0.52, Japan). Briefly, the morphological alterations were scored ranging from 0 to 3 (0 = all worms dead; 1 = minimal activity (severe reduction in motility), severe morphological/tegumental changes; 2 = slowed activity (reduced motility), first morphological/tegumental changes; and 3 = totally vital, normal activity, no morphological changes) [21]. Adult worms were considered dead when the movement was not observed for at least 2 minutes. The percentage of effect was calculated as described [21], and all experiments were carried in triplicate and presented as mean ± standard derivation (SD) values.

(1) $$\begin{array}{c} \%\text{Effect}=100-\frac{\left(\text{mean test}\right)*100}{\text{Mean control}}. \end{array}$$

### 2.4. Statistical Analysis

One-way analysis of variance (ANOVA) was used to evaluate significant differences between the means of the % effect of drugs and combinations. Statistical significance was set at a *P* value < 0.05.

## 3. Results and Discussion

Novel therapeutic approaches that not only eliminate the parasite but also could improve and ameliorate the pathologies associated with infection are required against schistosomiasis [16]. Repurposing and combination of drugs with active compounds presenting a different mode of action might be an interesting strategy [7]. We hypothesized that combining drugs with antioxidants not only could enhance the efficacy of the drug to eliminate the parasite but also could ameliorate disease sequelae associated with infection [16]. Previously, we evaluated several classes of drugs and antioxidants either alone or combined against newly transformed schistosomula (NTS) of *S. mansoni* and observed that several combinations were more active rather than drugs and antioxidant alone [17, 18]. Herein, we evaluated the efficacy of this novel therapeutic strategy against adult worms of *S. mansoni in vitro*.

### 3.1. Repurposed Drugs, Alone or Combined with Antioxidant Biomolecules, Showed Interesting Antischistosomal Activity

**Figure 2:**
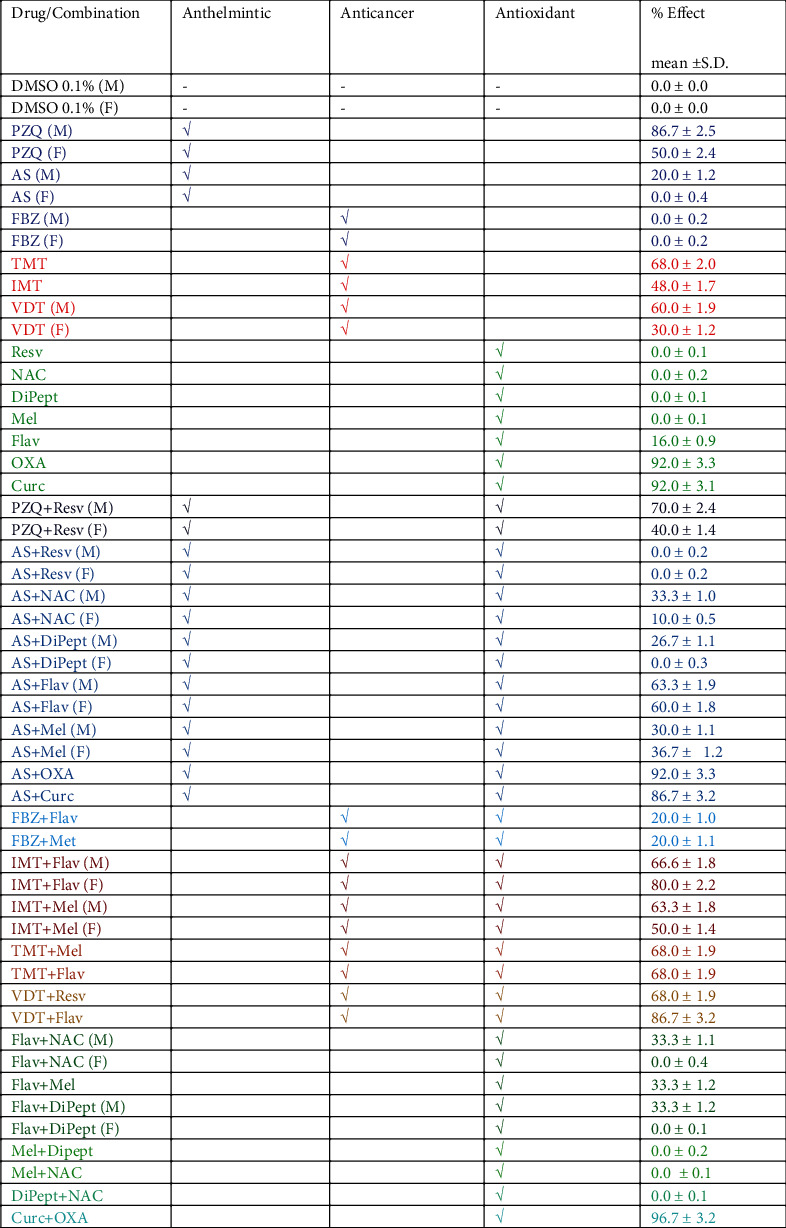
Score viability of the percentage of the effect of drugs and antioxidants against adult worms of *S. mansoni in vitro*. M: males; F: females

As depicted in Figure 2 and Supplementary Figure S1, the worms of the control group (RPMI 1640 with 0.1% DMSO) remained viable and without morphological alterations following 72 h postexposure. Regarding the anthelmintic drugs alone, PZQ was the most active with an effect of 87 ± 2.5% (ANOVA *P* = 0.15) on males and 50 ± 2.4% (ANOVA *P* = 0.12) on females. PZQ at 100 *μ*M induced the death of males following 24 h of exposure while female remains alive but with several morphological alterations after 72 h (Supplementary Figure S1 and Figure 2).

These results are aligned with those described in the literature reporting that males are more susceptible to PZQ than females [4]. In contrast, the males were less susceptible to AS than females as described elsewhere [22] which were dead following exposure for 48 h. Indeed, the effect of AS of 20.0 ± 1.2% (ANOVA *P* = 0.07) for females and 0.0 ± 0.4% (ANOVA *P* = 0.08) for males was lower than PZQ (Figure 2). Nevertheless, AS induced the decoupling at 48 h of exposure and death of the female by 72 h of exposure (Supplementary Figure S1 and Figure 2). Regarding the activity of PZQ and AS against NTS, PZQ induced severe morphological alterations on NTS, although most of the larvae survived following 72 h of exposure while AS induced the death of most larvae [17, 18]. These findings accorded with others that had shown that AS is more active against juvenile forms while PZQ is more active against adult worms [22]. The other anthelmintic drug, FBZ, has been reported that in mice reduce the number of adult parasites of *S. mansoni* [23]. However, FBZ did not affect our assays (0.0 ± 0.2% (ANOVA *P* = 0.17), Figure 2) on the morphology of males which remained vital and active (Supplementary Figure S1). The female worm seems to be more susceptible to drug presenting slight morphological changes (Figure 2 and Supplementary Figure S1). FBZ is more active against NTS than adult worms even more active than AS or PZQ [17]. FBZ may be of use during the initial stage of infection.

The antischistosomal activity of anticancer drugs was more pronounced against adult worms than the anthelmintics. TMT was most active (68.0 ± 2.0% (ANOVA *P* = 0.22)), followed by VDT (60.0 ± 1.9% (ANOVA *P* = 0.11) for males and 30 ± 1.2% (ANOVA *P* = 0.13) for females) and IMT (48.0 ± 1.7% (ANOVA *P* = 0.09), Figure 2). VDT and TMT induced the death of both parasites after 48 h of exposure (Figure 2). Following incubation in VDT, differences were apparent between the sensitivity of males and females; males were more susceptible than females, 60.0 ± 1.9% (ANOVA *P* = 0.07) and 30.0 ± 1.2% (ANOVA *P* = 0.10), respectively (Figure 2). IMT induced severe morphological alterations but not the death of parasites (Figure 2). Nonetheless, the decoupling of worms occurred following 17 h of exposure. Curiously, anticancer drugs also presented an antischistosomal activity against NTS [18] counteracting one of the major drawbacks of PZQ. These anticancer drugs are kinase inhibitors, and kinases play a pivotal role in key physiological processes including egg production [24]. The antischistosomal activity observed may derive from potential inhibition of these enzymes on parasites.

Of the antioxidants, OXA and Curc were active leading to the death of both sexes of the parasite following 17 h of exposure which translated in a percentage of effect > 90% (Figure 2). These results agreed with previous findings [25, 26]. OXA and Curc were more active than PZQ itself (Figure 2) in like fashion to what was seen with NTS [18]. Although the mechanism of action of these two antioxidants is uncertain, OXA might inhibit thioredoxin glutathione reductase (TGR) [25] while Curc could interfere with the ubiquitin-proteasome pathway [27]. Other reports suggest that Curc generates oxidative stress-inducing apoptosis and decreases the activity of antioxidant enzymes [28]. Recently, it has been demonstrated that Curc is effective not only against *S. mansoni* but also against *S. haematobium* [29]. Regarding the other antioxidants evaluated, Resv, NAC, DiPept, and Mel did not show antischistosomal activity against adult worms, as similarly noted for NTS [18]. The parasites incubated with these antioxidants remained viable without any morphological alterations during the assay (Figure 2; Supplementary Figure S1). Intriguingly, Resv displays moderate antischistosomal activity against NTS [18] while only slight morphological alterations were observed against adult worms, mainly on the females (Figure 2 and Supplementary Figure S1). On the other hand, Flav had a minimal effect (16.0 ± 0.9% (ANOVA *P* = 0.05), Figure 2) causing slight morphological alterations but not the death of parasites. In these cases, differences in susceptibilities between males and females were not apparent. By contrast, Flav exhibits moderate antischistosomal activity against NTS [18]. The mode of action for these two antioxidants remains unknown, although Resv may act on neuromotor activity [17, 18] and Flav may modulate key cellular enzymes [30]. Further studies are required to decipher the targets of antioxidants and drugs on adult forms of *S. mansoni*.

Herein, we evaluated the antischistosomal activity of combinations of antioxidants with broad-spectrum anthelmintic drugs. The antischistosomal effect in the combination of drugs with antioxidants was not different from those induced by drugs (e.g., TMT+Mel or TMT+Flav) or antioxidants alone (e.g., AS+OXA and AS+Curc) (Figure 2). Nevertheless, the antischistosomal activity observed for AS+NAC, AS+DiPept, AS+Flav, AS+Mel, IMT+Flav, FBZ+Flav or Mel, and VDT+Resv or Flav combinations was slightly better than drugs alone (Figure 2). The enhancement of the antischistosomal activity of IMT+Flav and VDT+Resv was more pronounced especially against females (Figure 2). While the combination of VDT+Flav induced a percentage of effect of 86.7 ± 3.2% (ANOVA *P* = 0.12), the drug alone has an effect of 60.0 ± 1.9% (ANOVA *P* = 0.16) for male and 30.0 ± 1.2% (ANOVA *P* = 0.11) for female (Figure 2).

On the other hand, combinations of PZQ+Resv and AS+Resv apparently act as an antagonist, especially against males. Male schistosomes incubated with these combinations presented a better viability score than those with drugs alone. This translated into a higher percentage of the effect of drug alone rather than combinations (Figure 2). These combinations against NTS were classified as synergistic [17]. These findings could suggest that the combination of the mode of action of PZQ or AS with Resv could be more effective against NTS than adult worms. Presumably, these combinations are more suitable for the initial stage of infection rather than chronic infection. By contrast, combinations as FBZ+Flav, IMT+Mel, or AS+Mel that act as slightly or antagonistic against NTS [18] enhance the antischistosomal effect in comparison to compounds alone (Figure 2). These findings indicated that development stages of parasites exhibit divergent susceptibilities which suggest that targets are different or a differentially expressed on larval and adult schistosomes.

Combinations of two antioxidants also were assessed. The antioxidants that presented slight or no antischistosomal activity when evaluated alone were combined (Figure 2). In general, these combinations did not improve their antischistosomal activity, most do not show any antischistosomal activity (Figure 2). Nevertheless, NAC, DiPept, and Mel combined with Flav displayed a moderate activity (33.3 ± 1.2% (ANOVA *P* = 0.08), Figure 2) especially against males (e.g., Flav+NAC and Flav+DiPept). These combinations slightly improved the activity of Flav alone, 33.3% vs. 16.0 ± 0.9% (ANOVA *P* = 0.10), Figure 2. Similarly, the two antioxidants were more active, i.e., Curc and OXA, when combined, inducing slightly increased activity against adult schistosomes (Figure 2).

### 3.2. Oviposition Was Markedly Affected by Antioxidants Alone or Combined with Drugs

**Figure 3:**
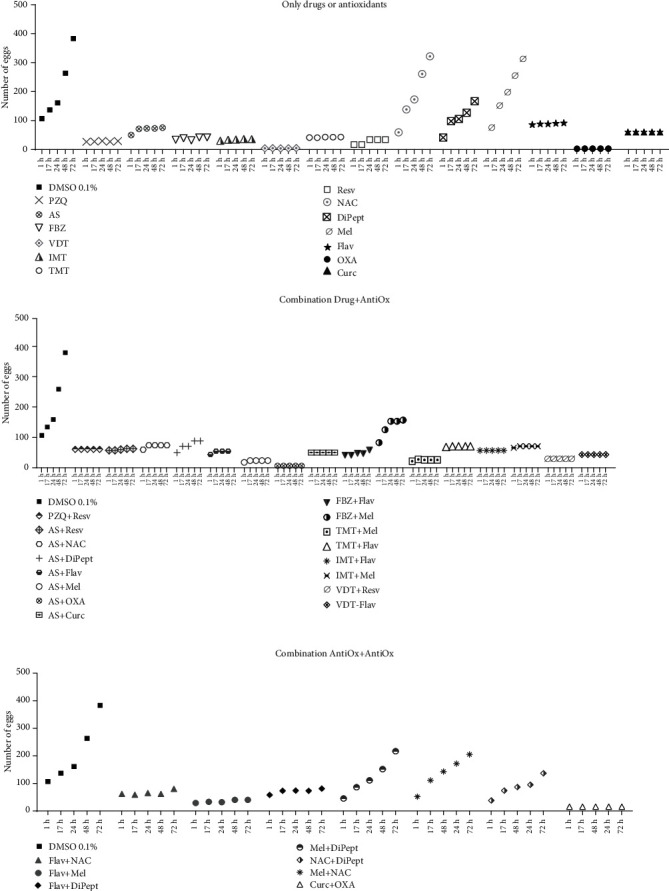
Number of eggs observed in vitro assay after drug/antioxidant application.

Chronic schistosomiasis is the consequence of continuous egg deposition that triggers inflammation, fibrosis, portal hypertension, and intestinal and pelvic organ-specific disease manifestations [31]. Fecal and urine egg excretions also are an indicator of worm viability. Thus, oviposition could be an important target for the development of novel approaches for schistosomiasis [32]. The reproductive capacity of parasites is based on two major criteria: pairing and egg production. The first indicate if the mating process occurs, and the latter is an indicator of egg output [33]. Although some compounds alone or combined did not induce the death of parasites, they could prevent oviposition which could be crucial to counteract the pathologies associated with infection (Supplementary Table S1 and Figure 3). All drugs evaluated against adult worms halted oviposition by the schistosomes either by inducing their death (e.g., PZQ) or by decoupling (e.g., AS). Notably, whereas FBZ did not induce severe morphological alterations or decoupling, it led to a cessation of the oviposition suggesting that the reproductive system of the female was affected. Indeed, the percentage of antischistosomal effect of FBZ was more pronounced in females rather than in males (Figure 2).

Concerning the antioxidants, OXA, Curc, Flav, and Resv alone also impeded oviposition (Figure 3). With OXA and Curc, this occurred due to the death of parasites. On the other hand, like FBZ, Flav and Resv did not induce severe morphological alterations or death of parasites (Figure 2) but the worms stopped releasing eggs (Figure 3). Even though these antioxidants did not induce severe morphological changes in adult worms, induced to the cessation of oviposition. This is a relevant fact since eggs release from parasites are linked to the formation of granuloma and inflammation associated with schistosomiasis, thus could be crucial to counteract the pathologies associated with infection. The mechanism of action of these antioxidants against schistosomes remains to be determined. Nevertheless, it may relate to the degenerative process of the female reproductive system, at least *in vitro*. In contrast, the other antioxidants evaluated (DiPept and NAC) did not affect oviposition or induce morphological alterations (Figure 2 and 3) reinforcing that they do not exert antischistosomal activity. However, all combinations of anthelmintic drugs with antioxidants also led to the cessation of oviposition similarly to that seen with compounds alone. This may be related to the activity of drugs or antioxidant alone, i.e., Flav and OXA, rather than the combination.

In combinations of antioxidant plus antioxidant, the cessation of oviposition only occurred when Flav was combined with other antioxidants (Figure 3), which might be related to the antischistosomal activity of Flav. The other combinations evaluated, e.g., Mel+DiPept, Mel+NAC, or NAC+DiPept, did not affect the oviposition. As noted, these antioxidants did not induce any morphological alterations which accord with the continuation of oviposition during the assay.

## 4. Conclusions

We report on the effect of the several classes of anthelmintic drugs and antioxidant biomolecules alone or combined against adult worms of *S. mansoni*. Not only antioxidants alone (e.g., OXA and Curc) were active against adult worms but also some combinations (e.g., anthelmintic drugs plus antioxidant or antioxidant plus antioxidant) enhanced antischistosomal activity. Nonetheless, the findings related to Curc should be interpreted carefully since this antioxidant is considered unstable and it is uncertain if the activity is related to a drug-like mechanism or indirect effects [34, 35]. The increased activity might be related to their different mode of action and/or targets on adult parasites. Since the schistosome eggs are the focus of the inflammatory process during schistosomiasis [31] and are implicated in carcinogenesis of the bladder during infection with *S. haematobium* [11], it might be useful to target oviposition in anthelmintic therapy. Indeed, the combination of different active agents evaluated during this study caused the cessation of the oviposition, at least *in vitro*. Considered together with the previous results with NTS [18], these present findings indicate that repurposing of anticancer drugs (or others) might be worthwhile since they were active on both larval and adult schistosomes [18]. Also, due to their biological properties of antioxidants in the prevention of DNA damage and blocking cancer initiation process [14, 15], they may be of use for the amelioration of disease sequelae including to impede carcinogenesis during urogenital schistosomiasis [11, 17]. Further studies using the best antioxidants (oxadiazole and curcumin) combined with drugs should be assessed *in vivo*.

## Data Availability

All data (structures of drugs, score viability, effect of drugs on oviposition, and morphological alterations of worms) used to support the findings of this study are included within the article and supplementary information file.

## Supplementary Materials

Supplementary Materials Table S1: oviposition expressed as the mean of number of eggs deposited per worm during *in vitro* assay. Figure S1: representative micrographs of adult worms following 72 h of exposure to anthelmintic drugs (praziquantel (PZQ), artesunate (AS), and flubendazole (FBZ)), anticancer drugs (imatinib (IMT), trametinib (TMT), and vandetanib (VDT)), antioxidants (4-phenyl-1,2,5-oxadiazole-3-carbonile,2-oxide (OXA), N-acetylcysteine (NAC), resveratrol (Resv), flavone (Flav), H-Trp-Ser-OH (DiPept), and melatonin (Mel)) alone, and combined (1 : 1) at 100 *μ*M. Viability score obtained during 72 h postexposure to compounds alone or combined.
