# Supplementary material for: Activity of Combinations of Antioxidants and Anthelmintic Drugs against the Adult Stage of Schistosoma mansoni
Source: J Parasitol Res. 2020 Aug 6;2020:8843808. doi: 10.1155/2020/8843808 (PMC7429017; doi:10.1155/2020/8843808)
Supplement: Supplementary Materials — Table S1: oviposition expressed as the mean of number of eggs deposited per worm during in vitro assay. Figure S1: representative micrographs of adult worms following 72 h of exposure to anthelmintic drugs (praziquantel (PZQ), artesunate (AS), and flubendazole (FBZ)), anticancer drugs (imatinib (IMT), trametinib (TMT), and vandetanib (VDT)), antioxidants (4-phenyl-1,2,5-oxadiazole-3-carbonile,2-oxide (OXA), N-acetylcysteine (NAC), resveratrol (Resv), flavone (Flav), H-Trp-Ser-OH (DiPept), and melatonin (Mel)) alone, and combined (1 : 1) at 100 μM. Viability score obtained during 72 h postexposure to compounds alone or combined. [file 8843808.f1.pdf]

Figure S1. Evaluation of anthelmintic drugs and drug combination on worms based on morphological alterations after 72 h post-exposure..

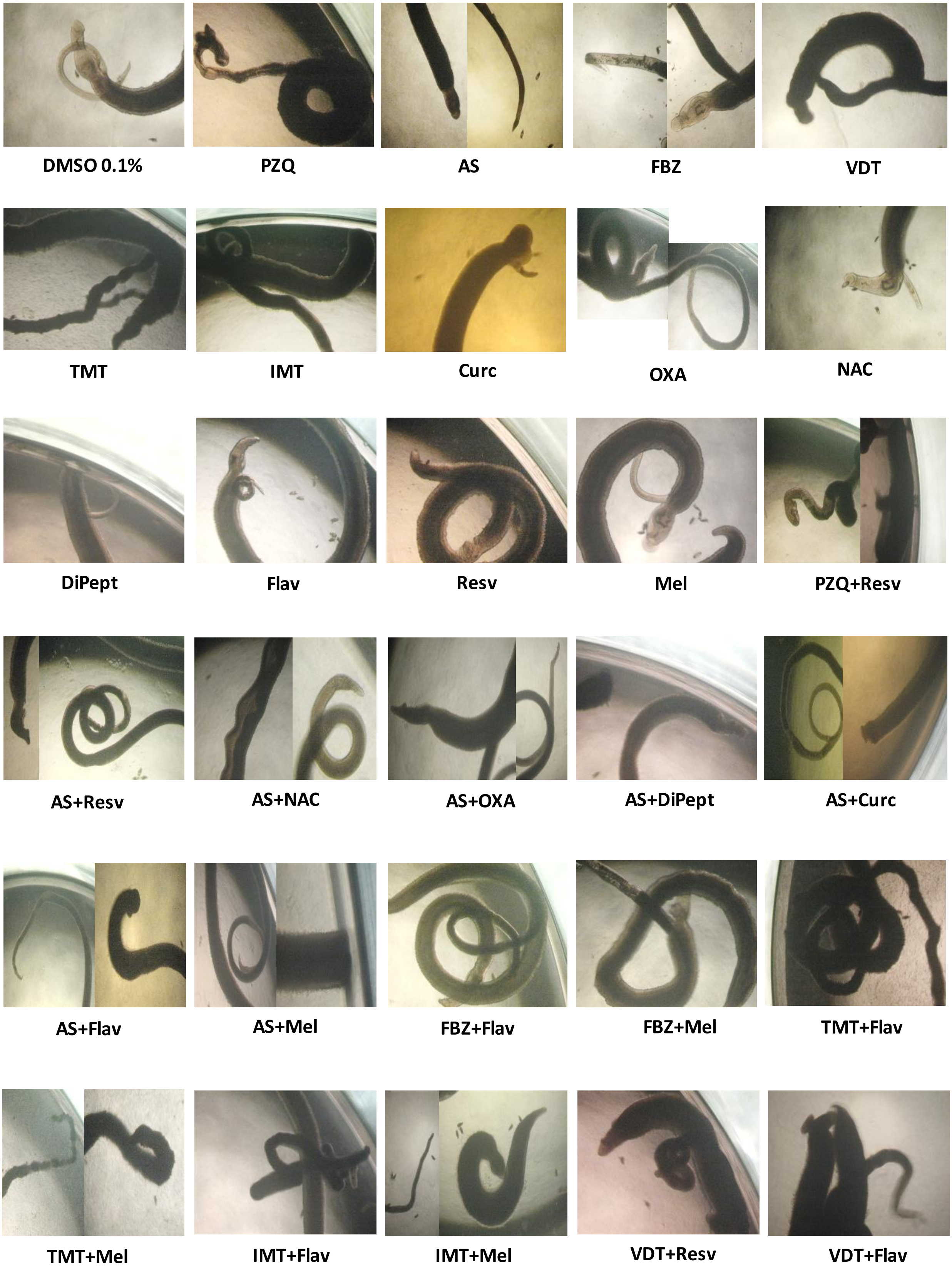

Table S1. Oviposition expressed as the number of eggs deposited per worm during in *vitro* assay.

	1h	17h	24h	48h	72h
	Mean ± SD	Mean ± SD	Mean ± SD	Mean ± SD	Mean ± SD
Compounds alone					
control	6.0 ± 0.0	29.3 ± 0.6	29.7 ± 0.6	70.7 ± 0.6	108.3 ± 2.3
DMSO 0.1%	107.0 ± 13.3	137.2 ± 39.2	161.5 ± 36.2	263.7 ± 4.7	383.5 ± 79.9
PZQ	26.7 ± 1.5	27.7 ± 1.5	29.7 ± 1.5	27.3 ± 2.1	29.3 ± 1.5
AS	50.3 ± 1.5	71.3 ± 1.5	73.3 ± 2.5	73.0 ± 2.0	75.7 ± 2.5
FBZ	33.7 ± 1.2	39.0 ± 1.7	31.7 ± 1.2	41.7 ± 1.5	41.7 ± 1.5
TMT	41.3 ± 0.6	41.0 ±1.0	43.0 ± 1.0	42.7 ± 1.2	42.7 ± 1.2
VDT	4.3 ± 1.5	4.7 ± 1.5	4.0 ± 1.0	4.3 ± 0.6	5.0 ± 2.0
IMT	30.7 ± 2.1	34.0 ± 2.0	34.7 ± 1.5	36.3 ± 1.5	36.0 ± 3.0
Resv	17.3 ± 0.6	17.3 ± 1.5	34.3 ± 3.1	34.3 ± 3.5	34.3 ± 3.1
NAC	59.3 ± 3.8	138.7 ± 2.5	173.0 ± 3.6	261.0 ± 2.0	322.0 ± 2.6
Flav	85.7 ± 0.6	88.0 ± 8.2	88.0 ± 8.2	88.0 ± 2.6	91.3 ± 1.5
Mel	69.7 ± 0.6	145.7 ± 3.5	192.3 ± 4.9	249.7 ± 1.5	307.0 ± 2.6
DiPept	41.3 ± 2.1	98.0 ± 1.0	105.7 ± 1.5	127.3 ± 2.5	167.0 ± 2.0
OXA	2.7 ± 0.6	3.0 ± 1.0	3.3 ± 0.6	3.3 ± 1.5	3.3 ± 1.5
Curc	52.7 ± 1.5	52.7 ± 1.5	52.7 ± 1.5	52.7 ± 1.5	52.7 ± 1.5
Drug+AntiOx					
PZQ+Resv	58.7 ± 1.5	60.3 ± 2.1	61.3 ± 1.5	61.0 ± 2.0	61.3 ± 1.5
AS+Resv	55.0 ± 2.0	56.3 ± 1.5	61.3 ± 2.3	61.7 ± 1.5	61.7 ± 2.3
AS+NAC	59.3 ± 2.1	75.3 ± 3.8	76.0 ± 2.6	76.3 ± 1.5	76.7 ± 1.5
AS+DiPept	50.7 ± 3.4	70.7 ± 1.2	72.3 ± 3.8	88.0 ± 1.0	88.0 ± 1.0
AS+OXA	4.7 ± 0.6	4.7 ± 0.6	4.7 ± 0.6	4.7 ± 0.6	4.7 ± 0.6
AS+Curc	49.3 ± 2.1	49.0 ± 2.6	49.0 ± 2.6	49.0 ± 2.6	49.0 ± 2.6
AS+Flav	43.3 ± 1.2	53.3 ± 1.2	53.3 ± 1.2	53.3 ± 1.2	53.3 ± 1.2
AS+Mel	16.7 ± 1.2	23.0 ± 1.0	23.0 ± 1.0	23.0 ± 1.0	23.0 ± 1.0
FBZ+Flav	41.7 ± 1.1	42.3 ± 2.1	47.7 ±1.2	47.0 ± 1.5	58.7 ± 4.0
FBZ+Mel	83.0 ± 2.0	127.0 ± 0.6	154.7 ± 4.9	155.3 ± 7.4	158.3 ± 1.2
IMT+Flav	57.7 ± 1.1	58.7 ± 1.2	57.0 ± 1.0	57.7 ± 1.5	57.3 ± 1.5
IMT+Mel	67.0 ± 1.0	71.7 ± 1.5	71.7 ± 1.5	71.7 ± 1.5	71.7 ± 1.5
TMT+Mel	22.3 ± 1.5	26.3 ± 2.1	25.0 ± 1.0	25.0 ± 1.0	25.0 ± 1.0
TMT+Flav	69.3 ± 0.6	71.0 ± 1.0	71.7 ± 1.5	71.7 ± 1.5	69.3 ± 1.2
VDT+Resv	28.0 ± 1.7	31.0 ± 1.7	31.0 ± 1.7	31.0 ± 1.7	33.7 ± 3.2
VDT+Flav	43.3 ± 2.1	43.3 ± 2.1	43.3 ± 2.1	43.3 ± 2.1	43.3 ± 2.1
AntiOx+AntiOx					
OXA+Curc	24.0 ± 1.7	24.0 ± 1.7	24.0 ± 1.7	24.0 ± 1.7	24.0 ± 1.7
Flav+NAC	61.7 ± 3.1	59.0 ± 1.0	65.0 ± 2.0	62.3 ± 0,6	80.3 ± 1.5
Flav+Mel	29.3 ± 1.2	32.7 ± 1.5	32.0 ± 2.0	40.3 ± 0,6	40.3 ± 2.5
Flav+DiPept	57.7 ± 0.6	72.7 ± 1.2	73.7 ± 0.6	73.0 ± 2.0	81.0 ± 2.6
Mel+Dipept	44.7 ± 1.5	86.0 ± 3.6	111.0 ± 3.0	152.0 ± 1.7	217.0 ± 9.8
Mel+NAC	52.3 ± 1.2	111.3 ± 0.6	143.0 ± 2.6	171.7 ± 1.2	205.0 ± 2.0
Dipept+NAC	38.0 ± 2.0	73.7 ± 0.6	86.7 ± 0.6	95.0 ± 1.0	137.3 ± 1.5
